# Adult onset pan-neuronal human tau tubulin kinase 1 expression causes severe cerebellar neurodegeneration in mice

**DOI:** 10.1186/s40478-020-01073-7

**Published:** 2020-11-23

**Authors:** Pamela McMillan, Jeanna Wheeler, Rachel E. Gatlin, Laura Taylor, Tim Strovas, Misa Baum, Thomas D. Bird, Caitlin Latimer, C. Dirk Keene, Brian C. Kraemer, Nicole F. Liachko

**Affiliations:** 1 Geriatrics Research Education and Clinical Center, Seattle Veterans Affairs Puget Sound Health Care System, S182, 1660 South Columbian Way, Seattle, WA 98108 USA; 2 Department of Psychiatry and Behavioral Sciences, University of Washington, Seattle, WA 98195 USA; 3 Department of Neurobiology and Anatomy, University of Utah, Salt Lake City, UT 84112 USA; 4 Science, Mathematics, Health, and Athletics Department, Northern Marianas College, Saipan, MP 96950 USA; 5 Department of Neurology, University of Washington, Seattle, WA 98195 USA; 6 Division of Medical Genetics, Department of Medicine, University of Washington, Seattle, WA 98104 USA; 7 Department of Laboratory Medicine and Pathology, University of Washington, Seattle, WA 98195 USA; 8 Division of Gerontology and Geriatric Medicine, Department of Medicine, University of Washington, Seattle, WA 98104 USA

**Keywords:** TTBK1, TTBK2, GABARAP, Cerebellum, Neurodegeneration, Spinocerebellar ataxia

## Abstract

The kinase TTBK1 is predominantly expressed in the central nervous system and has been implicated in neurodegenerative diseases including Alzheimer’s disease, frontotemporal lobar degeneration, and amyotrophic lateral sclerosis through its ability to phosphorylate the proteins tau and TDP-43. Mutations in the closely related gene *TTBK2* cause spinocerebellar ataxia, type 11. However, it remains unknown whether altered TTBK1 activity alone can drive neurodegeneration. In order to characterize the consequences of neuronal TTBK1 upregulation in adult brains, we have generated a transgenic mouse model with inducible pan-neuronal expression of human TTBK1. We find that these inducible TTBK1 transgenic mice (iTTBK1 Tg) exhibit motor and cognitive phenotypes, including decreased grip strength, hyperactivity, limb-clasping, and spatial memory impairment. These behavioral phenotypes occur in conjunction with progressive weight loss, neuroinflammation, and severe cerebellar degeneration with Purkinje neuron loss. Phenotype onset begins weeks after TTBK1 induction, culminating in average mortality around 7 weeks post induction. The iTTBK1 Tg animals lack any obvious accumulation of pathological tau or TDP-43, indicating that TTBK1 expression drives neurodegeneration in the absence of detectable pathological protein deposition. In exploring TTBK1 functions, we identified the autophagy related protein GABARAP to be a novel interacting partner of TTBK1 and show that GABARAP protein levels increase in the brain following induction of TTBK1. These iTTBK1 Tg mice exhibit phenotypes reminiscent of spinocerebellar ataxia, and represent a new model of cerebellar neurodegeneration.

## Introduction

Spinocerebellar ataxias (SCAs), a heterogeneous group of neurodegenerative movement disorders, affect an estimated 2.7 per 100,000 individuals worldwide [19]. Clinically, SCAs feature symptoms including progressive loss of balance and coordination, impairment of speech, abnormal eye signs, and characteristics of pyramidal tract dysfunction [11]. While not all SCAs have been characterized pathologically, those that have generally exhibit some level of atrophy of the cerebellum, including loss of cerebellar Purkinje cells, and degeneration of other connected central nervous system regions, usually in the absence of detectable pathological protein deposits. More than 40 different dominant mutations have been identified that cause SCA. At least 12 of these causative mutations are nucleotide repeat expansions in a variety of genes, while others are presumed loss- or gain-of-function changes in the affected gene.

Mutations in the gene encoding tau tubulin kinase-2, *TTBK2*, cause spinocerebellar ataxia, type 11 (SCA11). These mutations act in a dominant negative manner, and conditional knockout of mouse *Ttbk2* recapitulates phenotypes of SCA11 including loss of motor coordination and cerebellar degeneration [2–4, 7, 8]. Pathologically, SCA11 exhibits nearly complete loss of Purkinje cells and significant loss of cerebellar granule cells, accompanied by tau-positive neurofibrillary tangles, neuropil threads, and neurites in the hippocampus, substantia nigra, and other brain regions [8]. Another tau tubulin kinase, *TTBK1*, has also been implicated in neurodegenerative disease. Single-nucleotide polymorphisms (SNPs) in *TTBK1* are associated with a decreased risk of Alzheimer’s disease in a Han Chinese population, and may contribute to decreased neurofibrillary tangle formation and tau phosphorylation [26, 30]. TTBK1 has been shown to phosphorylate tau at Ser198, Ser199, Ser202, and Ser422 in paired helical filaments [21]. In *Drosophila*, co-expression of human TTBK1 and tau in adult neurons results in significant motor defects and shortened lifespan [6]. In mice, constitutive expression of full-length human TTBK1 results in impairment of spatial learning [22]. Co-expression of human TTBK1 with P301L mutant tau increases tau pathology including phosphorylation and aggregation, and drives locomotor dysfunction and loss of motor neurons in the spinal cord [29].

Both TTBK1 and TTBK2 are implicated in TDP-43 proteinopathies as well. In human tissue from patients with frontotemporal lobar degeneration (FTLD-TDP) or amyotrophic lateral sclerosis (ALS), TTBK1 is increased and co-localizes with TDP-43-positive inclusions. In mammalian cell culture and *C. elegans* models, the kinase domains of TTBK1 and TTBK2 phosphorylate Ser409 and Ser410 on TDP-43, epitopes consistently associated with TDP-43 proteinopathies including ALS and FTLD-TDP [15, 25].

Although TTBK1 and TTBK2 share a highly similar kinase domain, they have distinct accessory/regulatory domains and exhibit individual expression patterns, likely indicating diverged biological roles for these kinases [10, 24]. TTBK1 is present predominantly in the central nervous system, particularly in the cortex and cerebellum in adult brains, while TTBK2 is widely found in a variety of tissues including liver, muscle, pancreas, testes, and brain. In order to characterize the effects of high levels of TTBK1 in adult brains, we generated a transgenic mouse model expressing human TTBK1 under control of tetracycline operator (tetO) and the tetracycline transactivator (tTA) protein expressed from the pan-neuronal rodent neurofilament heavy polypeptide (NEFH) promotor; these bigenic mice are termed iTTBK1 Tg mice hereafter. When the tetracycline derivative doxycycline (DOX) is removed from the chow of iTTBK1 Tg mice, they accumulate full-length and truncated fragments of TTBK1 protein accompanied by spatial memory impairment, weight loss, neuroinflammation, and cerebellar neurodegeneration, culminating in average mortality around 7 weeks after transgene induction. These mice may be a model for studying the role of TTBK1 in neuronal health and disease, and may indicate a previously unknown role for TTBK1 in cerebellar disorders such as spinocerebellar ataxia.

## Materials and methods

### Mice

For this study, we generated the DOX-responsive B6.Cg-Tg(tetO-TTBK1)7Kra transgene, hereafter referred to as tetO-tau tubulin kinase 1 (tetO-TTBK1), by standard microinjection of a plasmid constructed by inserting the human full length TTBK1 cDNA (NM_032538.3) into exon 2 of the mouse prion protein promoter (pMoPrP) construct containing tetracycline operators in the PrP promoter essentially as previously described [20]. The driver transgene utilized was a pre-existing transgene obtained from The Jackson Laboratory (stock # 025397) carrying the human neurofilament heavy polypeptide (NEFH) promoter transgene B6.Cg-Tg(NEFH-tTA)8Vle/J (NEFH-tTA), which is inserted into chromosome 12: 6,917,892–6,917,912 without any disruption of gene regions. Animals carrying both tetO-TTBK1 and NEFH-tTA transgenes are referred to as iTTBK1 Tg mice. Their non-transgenic littermates (WT) and littermates carrying the NEFH-tTA single transgene were used as controls. Data from NEFH-tTA and WT mice were pooled to form the control group as there were no statistical differences between the two genotypes on any of the measures. To induce expression of the iTTBK1 transgene, mice were removed from DOX by switching from chow containing 625 ppm DOX (LabDiet or BioServ) to regular chow (5058/ PicoLab Mouse Diet 20). Mice were tested on the Barnes maze and Open field (33 iTTBK1 Tg, 16 WT, and 22 NEFH-tTA), and ranged from 3 to 5 months old at removal of DOX. Statistical analyses were performed using repeated measures ANOVA, with sex, genotype, and time since removal of DOX as independent variables. Sex was removed as a variable when no significant effects were detected. Post-hoc testing on individual time points was conducted by Tukey’s multiple comparisons test where appropriate.

### Weight and hindlimb clasping measures

Animals were weighed weekly beginning on the day DOX chow was removed, and their change in weight from their initial weight was calculated each week to monitor health. Hind limb clasping scores were also recorded weekly, with a score of 0 indicating no hind limb clasping, a score of 2 indicating fully clasped limbs, and a score of 1 indicating an intermediate phenotype. Once animals reached a clasping score of 2, they were provided with moistened chow on the floor of their cage to facilitate feeding.

### Inverted grid and grip testing

The ability to hang from an inverted wire grid, and the ability to grip a wire grid when pulled by the tail were used to examine limb strength. In the inverted grid test, mice were placed on a wire grid, gently pulled by the tail to ensure a grip, then the lid was inverted for a maximum of 1 min and the latency to fall from the grid was recorded. In the grip strength test, mice were placed on a horizontal wire mesh attached to a dynamometer so that all four paws gripped the wire, then the mouse was gently pulled away from the wire by its tail and the maximum force in grams was recorded. The inverted grid test was performed every week, while the grip test using the dynamometer was recorded every other week to avoid habituation to repeated testing.

### Open field testing

Open field testing was conducted in an opaque round arena 30 inches in diameter and occurred during the third week of transgene expression. All animals habituated in the testing room in their home cages for 45 min before the start of testing. Animals were free to roam the arena for 10 min and then were returned to their home cage. The apparatus was cleaned with 70% ethanol before the start of testing and between each animal. Distance traveled and time spent in each region was recorded using the ANY-maze tracking system (Stoelting Co).

### Barnes maze testing

Barnes maze testing began the day after open field testing and consisted of two training days, a rest day, and a probe day. A white plastic circle measuring 36 inches in diameter with twenty holes surrounding the edge was used for the Barnes maze. The escape hole contained a hidden box filled with clean bedding beneath the maze, which the mice had to locate and enter during testing. Additionally, large shapes placed along the edge of the maze served as spatial cues. Lighting was placed above and around the maze in order to achieve a light level of approximately 600 lx on the maze. Animals habituated in the room for 45 min before the start of testing. All animals received two training trials on two consecutive days, for a total of four training trials. Mice were placed in a clear cylindrical container for 10 s in the center of the maze before the start of each trial, then the container was removed and automated tracking (ANY-maze) began. During training trials, mice were free to roam the maze for 2 min. If mice located and entered the escape box before 2 min elapsed, then they were allowed to remain in the escape box for 30 s before they were returned to their home cage. Animals rested in their home cage for a minimum of 30 min between trials. If the animal did not locate the escape box during the allotted time, it was placed in front of the escape hole and allowed to enter the box and remain in it for 30 s. The time to locate and enter the escape hole was recorded during training trials. On the third day mice received a rest day and were not tested. On the final day of testing mice received a probe trial, in which the escape box was removed from the maze. During the probe trial mice were allowed to explore the maze for 2 min and the time to locate the escape hole recorded.

### Euthanasia criteria

After removal of DOX from the diet to induce transgene expression, mice were evaluated weekly until they reached euthanasia criteria of greater than 20% weight loss from their initial weight, a clasping score of 2, and an inverted grid hang time of less than 10 s. At this point, animals were euthanized by transcardial perfusion or CO_2_ asphyxiation, and fixed or fresh brains and spinal cords were taken. The dates of euthanasia of the iTTBK1 Tg animals were recorded and used to generate a survival curve.

### Ethics approval

All mouse experiments were reviewed and approved by the VA Puget Sound Health Care System Institutional Animal Care and Use Committee (IACUC) (Protocol #0884) and conducted in an American Association for Accreditation of Laboratory Animal Care (AAALAC)-accredited animal research facility.

### Tissue immunohistochemistry

Mice (8 WT, 7 NEFH-tTA, and 15 iTTBK1 Tg off dox for 1 month) were anesthetized and fixed by transcardial perfusion with 4% paraformaldehyde. Brains were removed and paraffin embedded for sectioning. Coronal sections were cut and stored at 4 °C until use. Mouse brain sections were deparaffinized and rehydrated through alcohols and an antigen retrieval step consisting of heat pretreatment by microwave or autoclave in citrate buffer was used when necessary. Sections were treated for endogenous peroxidases with 3% hydrogen peroxide in PBS (pH 7.4), blocked in 5% non-fat milk in PBS, and incubated with one of the following primary antibodies overnight at 4 °C: TTBK1 kinase domain (Clone F28701.1 from [22]), TTBK1 C-terminal domain (ProScience #5013), GFAP (Dako Z0334), Iba1 (Wako 019-19741), GABARAP (Abcam ab109364), AT8 (pS202/T205 tau, Thermo Scientific, #MN1020), pS422 Tau (Abcam #2866), pTDP (S409/410, clone 1D3) [16], and calbindin (Swant). Biotinylated secondary antibody was applied for 45 min at room temperature. Finally, sections were incubated in an avidin–biotin complex (Vector’s Vectastain Elite ABC kit, Burlingame, CA) and the reaction product was visualized with 0.05% diaminobenzidine (DAB)/0.01% hydrogen peroxide in PBS. Negative controls with secondary antibody alone did not immunostain tissue sections. Digital images were obtained using a Leica DM6 microscope with the DFC 7000 digital camera and LAS X imaging software. HALO digital image software (Indica Labs) was used to quantify GFAP, Iba1 and GABARAP immunoreactivity in the hippocampus. Brain sections were manually annotated around the regions of interest, average staining intensity for each antibody was determined to allow quantification without contribution of background staining, and a common threshold was then applied to all sections for that assay. Data represent the area of positive immunoreactivity within the region of interest divided by the total annotated area. This value was then multiplied by the average optical density of immunoreactivity to yield the final normalized IR area × OD. Data are displayed as mean ± SEM. A two tailed Student’s t-test was used to assess differences in immunoreactivity between mice overexpressing TTBK1 and controls. Purkinje cell loss was determined by assessing the number of calbindin stained Purkinje cells within a region of interest containing the 3Cb area.

### Tissue pathology

Comprehensive necropsy including examination of tissues from all major organs (heart, lungs, kidneys, adrenal glands, stomach, large and small intestine, pancreas, liver, spleen, thyroid, brain, spinal cord, and skeletal muscle) was performed. Briefly, after examination of the internal organs in situ, and removal and dissection and further gross examination individually, tissues were fixed in 10% neutral buffered formalin and processed for paraffin embedding and sectioning. Each paraffin tissue block was sectioned (5 µm thickness) and sections mounted on charged microscope slides and stained with hematoxylin and eosin. Histopathological examination was performed by a pathologist blinded to genotype.

### Yeast two-hybrid screen

TTBK1 bait plasmids were constructed in the pLexA-N vector under control of the LexA promoter: TTBK1 domain A (amino acids 297–733) and TTBK1 domain B (amino acids 775–1321). Cultures of TTBK1 bait plasmid yeast were grown overnight at 30 °C, brought to log phase the next day, and transformed with the Clontech Matchmaker Human Fetal Brain Library (1.3 mg/mL), a cDNA library in the pACT2 vector, resulting in greater than 1 × 10^6 transformants. Transformants were plated on 150 mm plates of SD minimal media lacking leucine, tryptophan, and histidine and containing 5–100 mM 3-Amino-1,2,4-Triazole (3-AT) and grown at 30 °C. Colonies were picked and grown in SD broth lacking tryptophan and leucine. The candidate prey plasmid from the matchmaker library was isolated, transformed and amplified in *E. coli*, re-isolated, and identified by sequencing from primers within the pACT2 backbone. The prey plasmid for positive, unique interacting proteins was then transformed back into L40 yeast along with either the TTBK1 bait or a MS2 plasmid. Four biological replicates of each transformation were struck onto SD minimal media lacking both tryptophan and leucine or SD minimal media lacking only tryptophan for controls and grown at 30 °C overnight. The colonies were then transferred onto nitrocellulose paper (GE Healthcare, Whatman Protran BA 85). The nitrocellulose was immersed in liquid nitrogen for 1 min and allowed to thaw for 2 min. The nitrocellulose was transferred to three layers of blotting paper that were saturated in Z buffer (Na_2_HPO_4_·7H_2_O, Na_2_HPO_4_·H_2_O, KCl, MgSO_4_·7H_2_O, adjusted to pH 7.0) with 5-bromo-4-chloro-3-indolyl-β-D-galactopyranoside (X-gal, Clontech Laboratories) dissolved in N,N-dimethylformamide (DMF) at 20 mg/ml, and sealed with parafilm within a petri dish. The plates were incubated at room temperature until the controls turned blue. Selective candidates displayed β-galactosidase activity prior to the controls, and did not show β-galactosidase activity with the MS2 plasmid.

### Immunoblotting

Protein samples were brought to 10 mM Tris, pH 6.8, 1 mM EDTA, 40 mM DTT, 1% SDS, 10% sucrose by addition of 5 × sample buffer boiled 5 min and loaded onto 4–15% pre-cast criterion SDS-PAGE gradient gels (Bio-Rad). To analyze protein aggregation and solubility, fractions were extracted with buffers of increasing solubilizing strength, using first low salt, then non-ionic detergents, ionic detergents and urea as previously described [17]. Antibodies used include TTBK1 kinase domain (Clone F28701.1 from [22]), TTBK1 C-terminal domain (ProScience #5013), Actin (clone AC40, Sigma A4700), and GABARAP (Abcam ab109364). Secondary goat anti-mouse or goat anti-rabbit IgG were the secondary antibody reagents used at a dilution of 1:1000 (GE Lifesciences). Immunoblot quantitation was performed using Image J software.

### Quantitative reverse transcription PCR

RNA was extracted from snap-frozen mouse hemi-brains using TRIzol Reagent according to the manufacturer’s recommendations [13]. To remove genomic DNA contamination, total RNA was treated with RQ1 RNase-free DNase (Promega) per manufacturer’s instructions. Reverse transcription was performed with iScript Reverse Transcription SuperMix (Bio-Rad) with a no-reverse transcriptase control for each sample (NRT). Quantitative PCR was performed using iTaq Universal SYBR GREEN Master Mix (BioRad) with NRT controls, no template controls, and a standard curve for each primer mix. Primers 5′ ATCCTGGAGTCCATTGAAGC 3′ and 5′ GCTAGCCCAAAGTCCAACAT 3 were used to detect TTBK1, 5′ TACCCTTCGTCTTGGGAAAC 3′ and 5′ CGAAGTTTGACGGTTTGATG 3′ to detect TTBK2, 5′ CCACAGGTGGCAAGTATGTC 3′ and 5′ GAAGATCTGGCCAAAAGGAC 3′ to detect normalizing gene TUBB5 and 5′ GCAGATGGCTCGAGAATACA 3′ and 5′ AAGCATTGGGGATCAAGAAC 3′ to detect normalizing gene YWHAZ.

## Results

### iTTBK1 Tg mice have robust inducible expression of human TTBK1

**Fig. 1:**
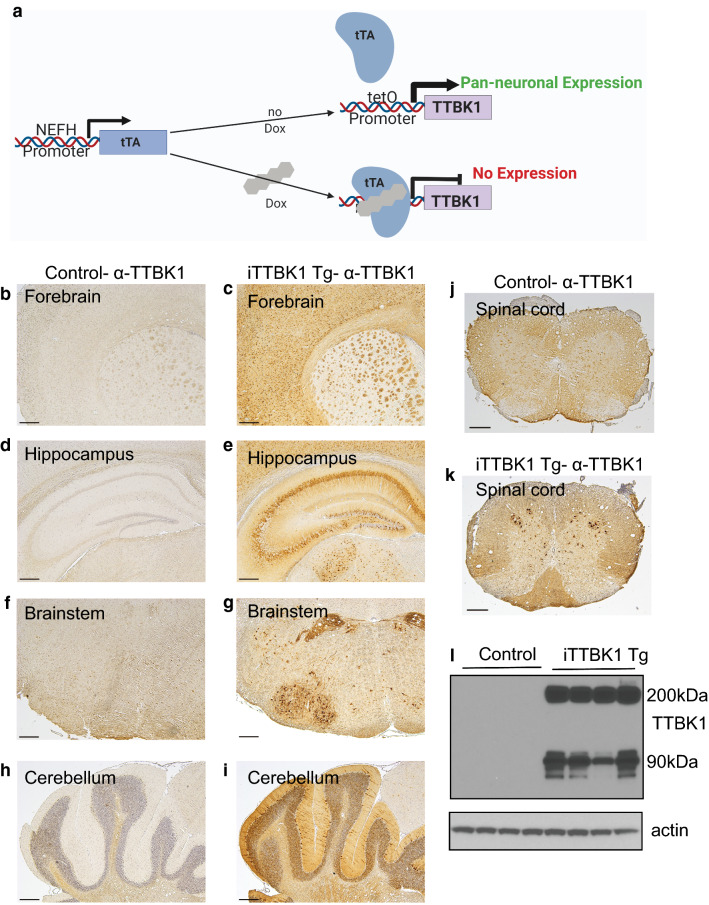
iTTBK1 Tg mice express high levels of human TTBK1. **a** Schematic of mouse transgene regulation. **b**–**k** Immunohistochemistry using an α-TTBK1 antibody raised against the C-terminus of the protein. Human TTBK1 is highly expressed in the adult mouse brain following induction of the iTTBK1 transgene. **b**, **d**, **f**, **h**, **j** Tissue from control mice. **c**, **e**, **g**, **i**, **k** Tissue from iTTBK1 Tg mice. Scale Bar = 500 µm. **b**, **c** Forebrain, **d**, **e** Hippocampus, **f**, **g** Brainstem, **h**, **l** Cerebellum, **j**, **k** Spinal cord. **l** Immunoblot of TTBK1 expression from control or iTTBK1 Tg whole brain lysate

The neuronally expressed kinase TTBK1 phosphorylates aggregation prone pathological proteins including tau and TDP-43 in the post-developmental central nervous system [15, 21, 25, 29]. However, it is unclear whether TTBK1 activity alone can drive neurodegenerative changes. To examine the consequences of increased TTBK1 activity in adult brain neurons, we generated mice with regulatable expression of human TTBK1 throughout the central nervous system driven by the NEFH promoter (Fig. 1a). These mice, termed inducible TTBK1 (iTTBK1 Tg), were first assessed for levels and expression of human TTBK1. Young iTTBK1 mice were born and bred in the presence of doxycycline (DOX). Young adult transgenic mice were removed from DOX exposure to induce iTTBK1 transgene expression and 1 month later brains were assessed by immunohistochemistry for increased TTBK1 immunoreactivity. Immunohistochemical analysis indicates a robust increase in TTBK1 protein throughout the brain, including the forebrain, hippocampus, brainstem and spinal cord (Fig. 1b–k and Additional file 1: Fig. S1a-b). Expression was confirmed by immunoblotting whole-brain or spinal cord lysate for TTBK1 (Fig. 1l and Additional file 1: Fig. S1c).

### iTTBK1 Tg mice exhibit progressive functional decline and shortened lifespan

**Fig. 2:**
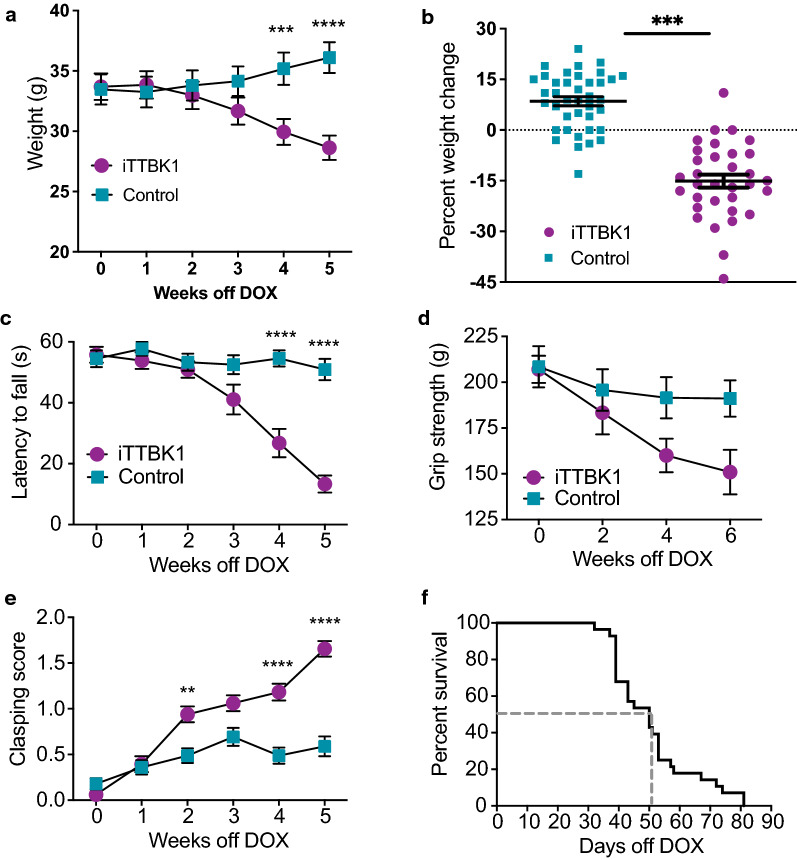
iTTBK1 Tg mice lose weight, exhibit motor dysfunction, and have decreased survival. **a** Change in weight over time of transgene induction in iTTBK1 Tg and control animals. Weight loss in iTTBK1 Tg mice began 2 weeks after removal from DOX and worsened progressively (mean ± SEM). ***p < 0.001; ****p < 0.0001. **b** Percent weight change from an individual animal’s initial weight while on DOX compared to their weight after 5 weeks of transgene expression. After 5 weeks after transgene induction iTTBK1 Tg animals showed marked decrease in weight. ***p < 0.001. **c** Latency of iTTBK1 Tg and control animals to fall from an inverted grid as a function of length of transgene expression (weeks off DOX). The iTTBK1 Tg mice showed decreasing latencies to fall over time, indicating progressive limb weakness (mean ± SEM). ****p < 0.0001. **d** Grip strength measurements in iTTBK1 Tg and control animals at 0, 2, 4, and 6 weeks off DOX using a dynamometer. Grip strength in iTTBK1 Tg mice declined with time after transgene induction (mean ± SEM), main effect of genotype: p = 0.069. **e** Increasing hind limb clasping behavior was observed in iTTBK1 Tg mice in the weeks following transgene induction (mean ± SEM). **p = 0.01, ****p < 0.0001. **f** iTTBK1 Tg mice have a shortened lifespan after transgene induction, with a median survival of 50 days. Control animals show no mortality during the scoring period (not plotted)

To evaluate phenotypes of iTTBK1 Tg mice, we tested a number of physical, functional, and behavioral characteristics over time. To assess weight changes over time, young adult mice were weighed weekly starting at their removal from DOX. Weight loss was observed in iTTBK1 Tg mice with significant differences from controls by week 3 after DOX removal (Fig. 2a and Additional file 2: Fig. S2a, b). iTTBK1 Tg animals weighed significantly less than controls (main effect of genotype, p = 0.004). Additionally, animals lost significant weight as the length of transgene expression increased driven by the progressive weight loss in iTTBK1 Tg animals after the removal of DOX (main effect of week, p < 0.001; week × genotype interaction p < 0.001). After 5 weeks of transgene expression, iTTBK1 Tg animals had lost a greater percentage of their initial weight, compared to control animals (Fig. 2b and Additional file 2: Fig. S2c, d, main effect of genotype, p < 0.001).

To assess changes in muscle strength, we used two assays, the inverted grid test and grip strength measurement using a dynamometer. On the inverted grid test iTTBK1 Tg animals exhibited a significant decreased latency to fall, compared to controls (Fig. 2c and Additional file 2: Fig. S2e, f, main effect of genotype, p < 0.001). Animals also had decreased latency on the inverted grid at later time points after transgene induction, driven by the progressively decreasing latency to fall in iTTBK1 Tg animals (main effect of week, p = 0.01; week × genotype interaction, p < 0.001). iTTBK1 Tg animals showed a trend towards decreased strength that approached but did not achieve statistical significance (Fig. 2d and Additional file 2: Fig. S2g-h, main effect of genotype, p = 0.07).

We examined hind limb clasping in iTTBK1 Tg mice as a measure of neurological changes over time. We found that iTTBK1 Tg mice had higher average clasping scores compared to controls, and exhibited progressive hind limb clasping (Fig. 2e and Additional file 3: Fig. S3a, b, main effect of genotype, p < 0.001). Additionally, iTTBK1 Tg animals tended to have higher clasping scores at later time points (main effect of week, p < 0.001; week × genotype interaction, p < 0.001).

To estimate survival after TTBK1 induction, iTTBK1 Tg mice were weighed and examined for hind limb clasping weekly after transgene induction, and wire hang measurements were taken every other week until animals reached the following three endpoint criteria: greater than 20% weight loss from their initial weight, a clasping score of 2, and a wire hang time of less than 10 s. The median survival of iTTBK1 Tg animals was 50 days after transgene induction, with a maximum survival 81 days after transgene induction (Fig. 2f). No control animals reached endpoint criteria during this time period.

### iTTBK1 Tg mice have impaired spatial memory

**Fig. 3:**
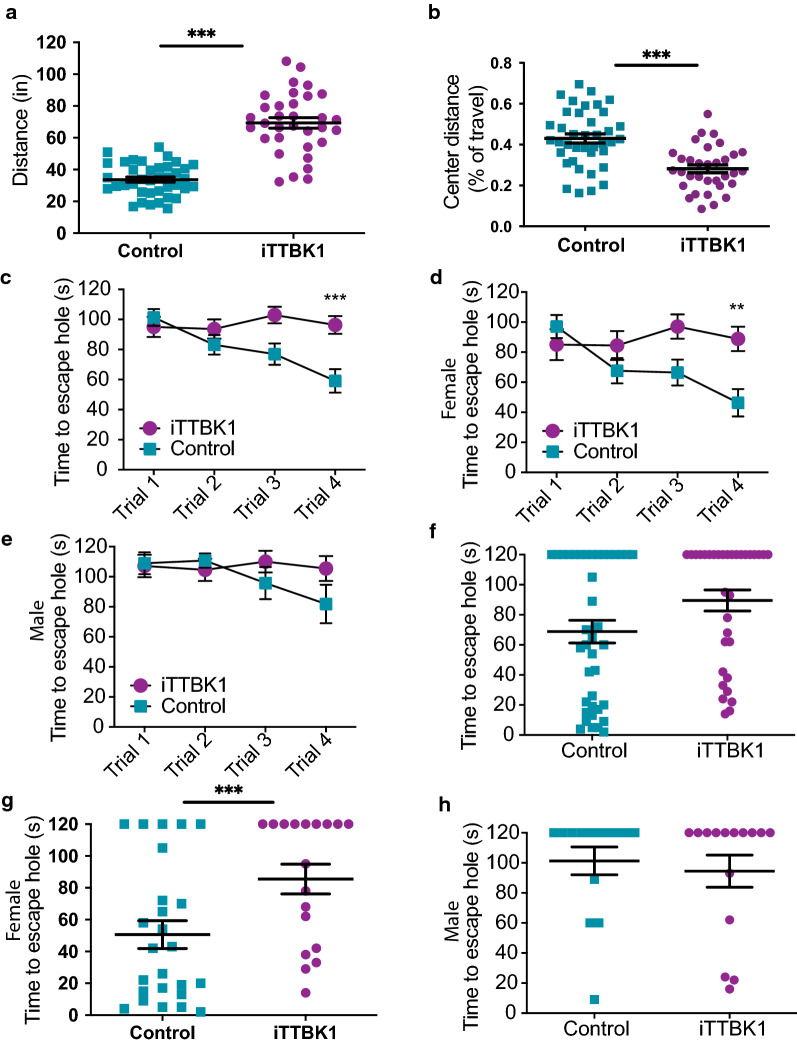
iTTBK1 Tg mice exhibit hyperactivity and impairments in spatial memory. **a** Open field testing of control and iTTBK1 Tg mice. iTTBK1 Tg are hyperactive compared to controls, ***p < 0.001. **b** Open field testing of control and iTTBK1 Tg mice. iTTBK1 Tg travel less in the center of the arena compared to controls. Percentage of distance travelled in the center of the field versus the periphery, ***p < 0.001. **c** iTTBK1 Tg mice have impaired learning of the location of the escape hole during Barnes maze training trials (mean ± SEM), ***p = 0.001. In addition to the main effect of genotype (p = 0.046), there was also a significant main effect of sex (p = 0.001). **d** Female control mice learn the location of the escape hole during Barnes maze training, while iTTBK1 Tg female mice do not, **p = 0.02. **e** Both male control and iTTBK1 Tg mice fail to learn the location of the escape hole during Barnes maze training (trial #4: p = 0.52). **f** On the day of the Barnes maze probe trial, iTTBK1 Tg mice do not exhibit significant impairments in locating the escape hole (main effect of genotype, p = 0.16). However, as in the training trials, there were significant sex effects (main effect of sex, p = 0.003; genotype × sex interaction, p = 0.04). **g** On the day of the Barnes maze probe trial, female control mice are able to find the escape hole significantly faster than female iTTBK1 Tg mice, ***p = 0.007. **h** On the day of the probe trial, male control and iTTBK1 Tg mice exhibit similar times to find the escape hole

The iTTBK1 Tg animals were evaluated for motor function using the open field test to measure locomotor activity. In the open field test, iTTBK1 Tg animals were more active than controls (Fig. 3a and Additional file 3: Fig. S3c, d, main effect of genotype, p < 0.001), but this activity was shifted towards the periphery of the arena rather than the center (Fig. 3b and Additional file 3: Fig. S3e, f, main effect of genotype, p < 0.001). The high activity of iTTBK1 Tg animals in the open field also serves as a control for analysis of learning and memory performance as measured by the Barnes maze; severe deficits in open field performance can confound Barnes maze performance by limiting subject’s ability to move about the maze and learn the task. The Barnes maze was then used to test spatial memory. During the training days on the Barnes maze, control animals located the escape hole faster than iTTBK1 Tg animals (Fig. 3b, main effect of genotype, p = 0.046), and females located the escape hole more quickly than males (Fig. 3c, d, main effect of sex, p < 0.001). Overall, animals located the escape hole in significantly less time in later trials (main effect of trial, p = 0.004). However, this effect was driven by control animals locating the escape hole more quickly in later trials than the iTTBK1 Tg animals (trial × genotype interaction, p < 0.001). In the probe trial, there was no difference in the time to locate the escape hole between the two genotypes (Fig. 3e, main effect of genotype, p = 0.16), although there was a significant main effect of sex (p = 0.003). Some animals failed to learn this task; however, these were present in both controls and iTTBK1 Tg mice. Despite these animals, we were able to detect a significant difference between genotypes. Post-hoc testing revealed that female control animals performed significantly better in the probe trial than did the female iTTBK1 Tg animals (Fig. 3f, p = 0.007), but there was no significant difference between the genotypes among male animals (Fig. 3g).

### iTTBK1 Tg mice exhibit neuroinflammation and neurodegeneration in the absence of pathological protein accumulation

TTBK1 phosphorylates both TDP-43 and tau and has been implicated in proteinopathies that feature these inclusions, including FTLD and ALS. Previous data from our lab found increased levels of TTBK1 in FTLD cases compared to normal controls [15], suggesting changes in TTBK1 protein abundance or processing may be a contributor to disease. To assess whether expression of human TTBK1 leads to neuropathological changes, adult mice were removed from DOX to induce the transgene and 1 month later brains were assessed by immunohistochemistry. We first assessed whether iTTBK1 Tg expression would promote phosphorylation of endogenous mouse tau or TDP-43. However, immunoblotting or immunostaining for phospho-tau (AT8, AT180, phospho-Ser 422) and phospho-TDP-43 (phospho-Ser 409 and 410) failed to demonstrate these pathological species in our mouse model (Additional file 4: Fig. S4a-g) despite robust induction of TTBK1 protein. In addition, there was no evidence of hippocampal neuronal loss when assessed for NeuN (Additional file 4: Fig. S4h, i.). To determine whether there was accumulation of insoluble TTBK1, we performed serial sequential extractions with detergents of increasing solubilizing strength. We found accumulation of detergent-insoluble TTBK1, with the higher-molecular weight isoform being more present in insoluble fractions (Additional file 5: Fig. S5a).

**Fig. 4:**
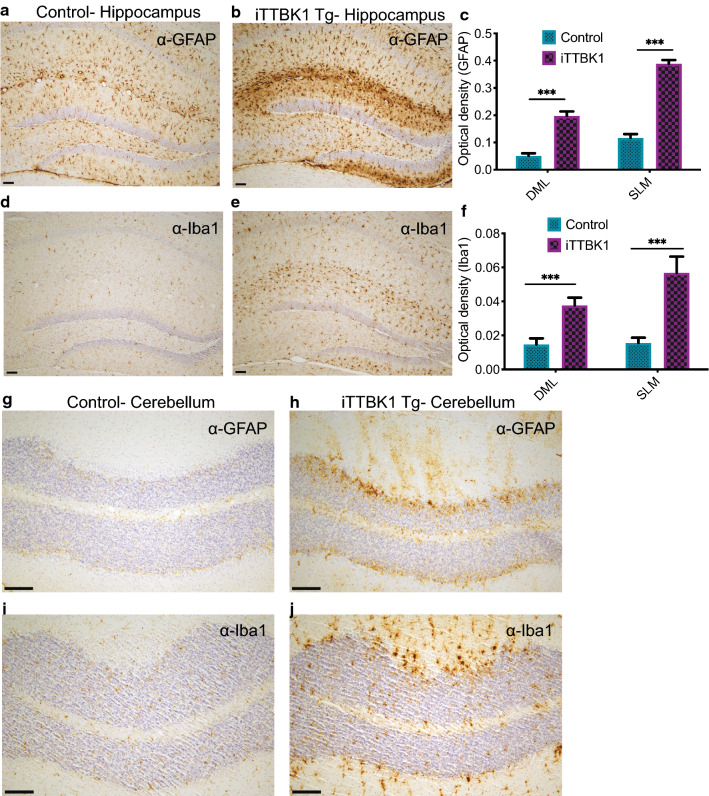
iTTBK1 Tg mice exhibit increased neuroinflammation. **a**–**f** Representative hippocampal brain sections from control and iTTBK1 Tg mice immunostained for GFAP (**a**, **b**) and Iba1 (**d**, **e**). Scale bar = 100 μm. **c**, **f** Induction of the transgene in iTTBK1 Tg mice significantly increased GFAP and Iba1 immunoreactivity in the hippocampus. ***p < 0.0001 by two-tailed t test. *DML* dentate molecular layer, *SLM* stratum laculosum moleculare. **g**–**j** Representative immunostaining of GFAP and Iba1 in the cerebellum was increased in all iTTBK1 Tg animals compared to control animals. **g**, **h** GFAP, **i**, **j** Iba1 Scale Bar = 200 μm. Immunostaining of GFAP and Iba1 in the cerebellum was increased in all iTTBK1 Tg animals compared to control animals but the signal was too diffuse to quantify

**Fig. 5:**
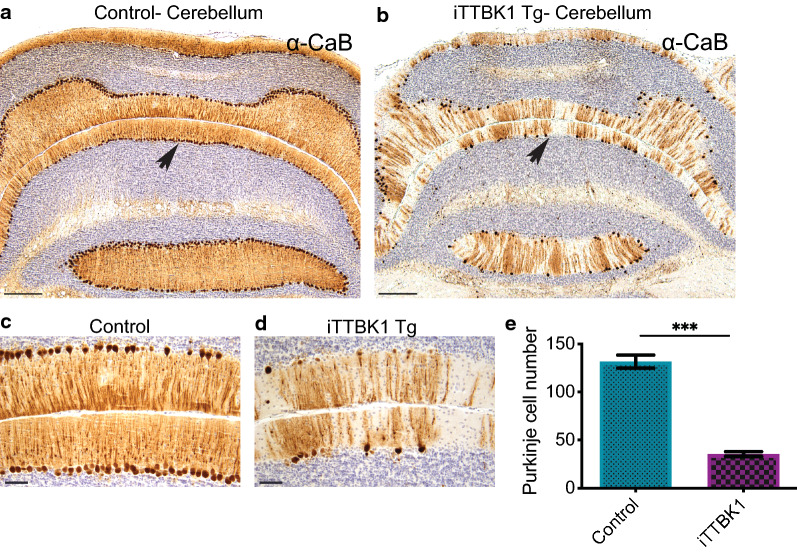
iTTBK1 Tg mice exhibit dramatic Purkinje cell loss. **a**, **b** Representative low-power images of CaB immunostaining in the cerebellum of control and iTTBK1 Tg animals. iTTBK1 Tg animals demonstrate wide scale Purkinje cell loss throughout the cerebellar lobules. Arrows indicate the cerebellar lobule depicted at higher power in **c** and **d** where Purkinje cell number was quantified (**e**). ***p < 0.0001 by two-tailed t test. Scale Bar = 500 μm (**a**, **b**) and 100 μm (**c**, **d**)

Despite the lack of pathological phospho-tau and TDP-43 in brains of our transgenic mice, these mice exhibited deteriorating health and motor impairment, resulting in early achievement of criteria for euthanasia. To determine the potential cause of iTTBK1 Tg animal decline, we performed necropsies including every major organ system. Histopathological examination of cardiopulmonary, gastrointestinal, hematolymphatic, genitourinary, renal, neuromuscular, and neuroendocrine systems, among others, failed to identify any diagnostic abnormalities, including no evidence of developmental abnormalities, infection, inflammation, or neoplastic processes. However, examination of the brains of these animals demonstrated robust increases in GFAP and Iba1 in the hippocampus, indicating both astrogliosis and innate immune activation (Fig. 4a–f) in this region despite normal appearing neurons in normal density in the hippocampus. This neuroinflammatory response was also evident in the cerebellum, especially in the Purkinje cell layer (Fig. 4g–j and Additional file 5: Fig. S5b, c) where we also detected dramatic evidence of Purkinje cell drop out in the cerebellum of iTTBK1 Tg mice (Fig. 5a–e). This Purkinje cell loss was evident throughout all lobules in the anterior lobe. When the number of Purkinje cells was quantified in lobule 3 of the cerebellum, we detected a decrease of 73% in iTTBK1 Tg mice compared to control animals (Fig. 5e). Cerebellar Purkinje cell loss could readily explain pronounced motor and cognitive phenotypes observed in iTTBK1 Tg mice. We did not find significant alterations in primary motor and sensory cortex or other areas of cerebral cortex and white matter. Brainstem and spinal cord neurons and fiber tracts were intact without significant histopathologic abnormalities.

### Expression of human TTBK1 in mouse neurons does not affect endogenous mouse *Ttbk2*

Loss of mouse *Ttbk2* causes Purkinje cell and cerebellar neurodegeneration reminiscent of that observed in iTTBK1 Tg mice and in SCA11 [3]. Therefore, we tested whether expression of human TTBK1 in iTTBK1 Tg mice decreased the levels of mouse *Ttbk2* mRNA in the brain using quantitative reverse-transcription PCR (qRT-PCR) (Additional file 5: Fig. S5d). We found that mRNA levels of *Ttbk2* are unchanged between control and iTTBK1 Tg mice, indicating phenotypes observed in iTTBK1 Tg mice are driven by expression of TTBK1 rather than loss of TTBK2.

### iTTBK1 Tg exhibit elevated levels of GABARAP, a novel TTBK1 binding partner

**Fig. 6:**
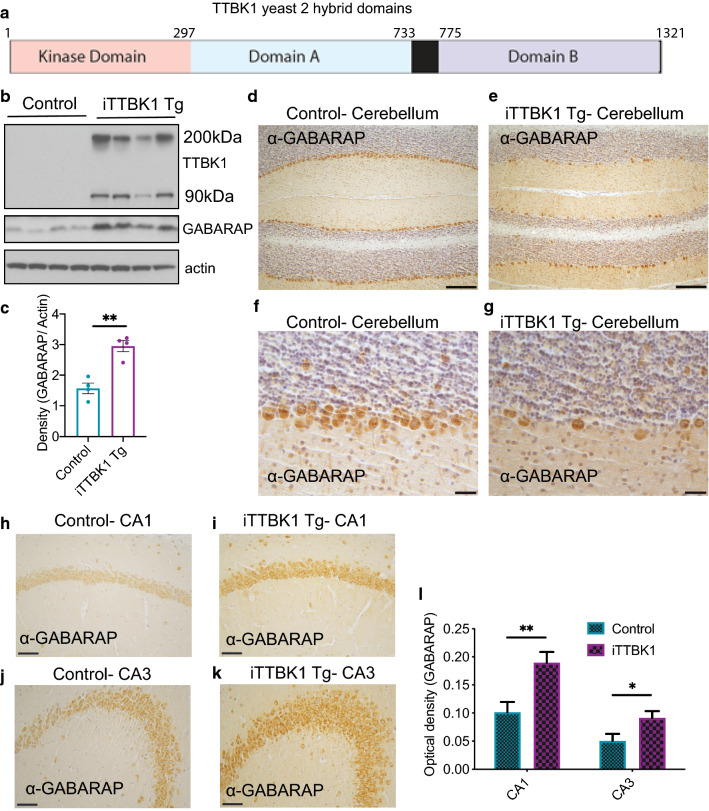
GABARAP binds TTBK1 and is increased in iTTBK1 Tg mice. **a** TTBK1 protein domains used during yeast-2-hybrid assay. **b**, **c** GABARAP has increased protein expression in iTTBK1 Tg mouse brains detected by immunoblot. Quantitation of immunoblots in (**c**), **p = 0.0015, unpaired t-test. **d**–**g** Representative brain sections from control and iTTBK1 Tg mice immunostained for GABARAP in the cerebellum. (**d**, **f**) Control animals, (**e**, **g**) iTTBK1 Tg. (**d**, **e**) Scale bar = 250 µm. (**f**, **g**) Scale bar = 50 µm. **h**–**k** Representative brain sections from control and iTTBK1 Tg mice immunostained for GABARAP in the hippocampus. **l** GABARAP expression increases in mouse hippocampal CA1 (**h**, **i**) and CA3 (**j**, **k**) pyramidal neurons following induction of TTBK1. **p = 0.0025 and *p = 0.02 by two-tailed t test. Scale Bar = 100 µm

Despite cognitive, motor, neuroinflammatory and neurodegenerative phenotypes in iTTBK1 Tg, there is no apparent tau or TDP-43 neuropathology. Therefore, increased TTBK1 may promote dysregulation of another unknown interactor that contributes to these phenotypes. To identify interacting protein partners of TTBK1, we performed a yeast-2-hybrid screen using the central protein interaction domain or the C-terminus of TTBK1 as bait (Domains A and B, Fig. 6a). Several candidate TTBK1 interacting partners were identified in this screen (Table 1), including members of the Gamma-aminobutyric Acid Receptor–Associated Proteins (GABARAP) family (GABARAP and GABARAP L2), which are implicated in GABA receptor transport, intra-golgi trafficking, and autophagosome maturation [23]. To test whether GABARAP protein expression is altered in response to increased TTBK1, we examined GABARAP immunoreactivity in the brains of iTTBK1 mice by immunoblot and immunohistochemistry analyses. GABARAP immunoreactivity was increased in total brain tissue (Fig. 6b, c). Immunohistochemistry of GABARAP in the cerebellum found robust expression in Purkinje cells in both control and iTTBK1 Tg animals (Fig. 6d–g). Interestingly, in the hippocampus, GABARAP expression is increased (Fig. 6h–l). This may indicate region specific upregulation of GABARAP in response to TTBK1 overexpressing. Taken together, these results suggest GABARAP and TTBK1 may have a functional in vivo relationship.

**Table 1:** Proteins that interact with TTBK1

TTBK1 Bait Region	Interacting gene	Protein name	Cellular function
Domain A	GABARAP	Gamma-aminobutyric Acid Receptor—Associated Protein	GABA receptor transport, autophagosome maturation
	GABARAPL2	Gamma-aminobutyric Acid Receptor—Associated Protein	Intra-golgi trafficking, autophagosome maturation
	ITSN2	Intersectin 2	Endocytic membrane trafficking, microtubule assembly, dendrite formation
	PTPRK	Protein-Tyrosine Phosphatase Kappa	Cell contact and adhesion
Domain B	SMS	Spermine Synthase	Spermine to spermidine catalysis
	XRCC6	X-ray Repair Cross Complementing Protein 6	Helicase, DNA damage repair, innate immune response

## Discussion

We have developed a novel model of inducible human TTBK1 overexpression in the murine central nervous system. Following induction, these mice exhibit progressive weight loss, decreased motor function, cognitive impairment, and shortened lifespan. Pathologically, soluble and insoluble TTBK1 accumulates throughout the brain and spinal cord, accompanied by increased neuroinflammation and dramatic loss of cerebellar Purkinje cells. We did not observe an increase in phosphorylation or aggregation of endogenous mouse tau or TDP-43, which are previously identified targets of TTBK1 [15, 21, 25, 29]. In the course of this work, we identified novel TTBK1 binding partners, including GABARAP, GABARAPL2, Intersectin 2 (ITSN2), Protein Tyrosine Phosphatase Receptor Type κ (PTPRK), Spermine Synthase (SMS), and X-ray Repair Cross Complementing 6 (XRCC6), and found that GABARAP expression increases in iTTBK1 Tg.

GABARAP and GABARAPL2 are mammalian orthologs of the yeast autophagy-related protein Atg8, which controls autophagosome expansion and cargo recruitment [23]. Both GABARAP and GABARAPL2 are required for autophagosome-lysosome fusion through interactions with the vesicle fusing ATPase NSF, and have roles in autophagy mediated anti-viral defense and mitophagy. GABARAP and GABARAPL2 have been previously implicated in neuron health and function. GABARAP interacts with both microtubules and GABA_A_ receptors, and traffics GABA_A_ receptors to the neuronal plasma membrane [14, 18]. GABARAP is present throughout the cerebral cortex, hippocampus, and cerebellum, and enriched in the axonal initial segment (AIS) of hippocampal pyramidal neurons and cerebellar Purkinje cells [12]. Selective deletion of the autophagy gene *Atg7* in adolescent mouse forebrain GABAergic interneurons results in behavioral deficits accompanied by aggregation and mislocalization of GABARAP and GABARAPL2, reduced surface GABA_A_ receptors, altered GABA_A_ receptor trafficking, and increased GABA release from interneurons [9]. Although we have shown that GABARAP and TTBK1 can interact by yeast-2-hybrid, the relationship between GABARAP and TTBK1 in neurons remains to be defined, including whether there are cerebellar specific aspects of their interaction. It is possible that dysregulation of GABARAP functions either through alterations in GABA_A_ receptor dynamics or neuronal autophagy mediates the behavioral and neuropathological phenotypes observed in iTTBK1 mice. We found sex-specific differences in behavior of iTTBK1 Tg mice in open field and Barnes maze behavioral tests; however, the basis for these differences are currently unknown. Future work will be needed to determine the roles of TTBK1 or GABARAP in these phenotypes.

While prior modeling experiments would suggest that TTBK1 activity is upstream of tau and TDP-43 phosphorylation, we did not observe an increase in these phospho-epitopes in the iTTBK1 Tg mice. Instead, we found that TTBK1 is capable of driving neuronal dysfunction, neuroinflammation, and neurodegeneration independent of pathological tau or TDP-43. Immunoblot analysis of soluble and insoluble TTBK1 identified a number of protein species, which represent full-length and truncated forms. The presence of doublets in the 90 kDa range may indicate post-translational modifications including phosphorylation as TTBK1 autophosphorylation has recently been shown to modulate its enzymatic activities [1]. It is currently unknown whether solubility or post-translational modifications of TTBK1 affect phenotypes in this model.

While we looked for a cause of the rapid decline in weight and motor dysfunction observed in iTTBK1 Tg mice, there were no apparent organ or tissue changes that would suggest an alternative beyond neuronal dysfunction or neurodegeneration. It is currently unknown why cerebellar Purkinje cells are selectively vulnerable to TTBK1 expression. It is possible that similar to TTBK2, TTBK1 has roles in maintenance of primary cilia that may contribute to these phenotypes [3]. Mutations in the kinase TTBK2 cause SCA11, and conditional knockout of mouse *Ttbk2* during adulthood results in motor coordination deficits and cerebellar degeneration including loss of Purkinje cells [3, 8]. Phenotypes of iTTBK1 mice are reminiscent of loss of *Ttbk2* gene expression; therefore, we tested whether induction of TTBK1 resulted in a decrease in *Ttbk2* mRNA. However, we did not see any change in expression of *Ttbk2*, indicating that TTBK1 may have a direct role in potentiating neuronal dysfunction and neurodegeneration. There is some evidence supporting this idea. In *Drosophila* neurons, constitutive expression of human TTBK1 is profoundly toxic and results in larval lethality, while inducible expression of TTBK1 during adulthood causes motor deficits and shortened lifespan [6]. Although TTBK1 has been shown to exacerbate tau or TDP-43 neurotoxicity, this may occur downstream or in parallel to phosphorylated protein accumulation, and our data suggest that TTBK1 activation directly causes neurodegeneration.

The phenotypes of iTTBK1 mice, which include progressive weight loss, impaired movement and cognition, and cerebellar neurodegeneration, are reminiscent of human spinocerebellar ataxias. These disorders are likewise characterized by significant decline in body weight, impaired balance and coordination, damage to cerebellar Purkinje neurons, and cerebellar atrophy [5, 11]. In addition to providing a system to study the neuronal functions of TTBK1, iTTBK1 mice may represent a new model for spinocerebellar ataxia or other disorders characterized by cerebellar dysfunction and degeneration. Our data suggest that TTBK1 misregulation might cause human disease, and nominate the human TTBK1 gene as a potential target for gain of function mutations as a possible cause of genetically unexplained SCAs.

## Supplementary information

Additional file 1: Figure S1. **a**, **b** Immunohistochemistry using an α-TTBK1 antibody raised against the C-terminus of the protein. Scale bars = 50 µm (**a**) Hippocampus CA3 region. (**b**) Cerebellar Purkinje cells. **c** Immunoblot of TTBK1 expression from control or iTTBK1 Tg spinal cord lysate.

Additional file 2: Figure S2. Weight, inverted grid, and grip strength data separated by sex. **a**, **b** Change in weight over time of transgene induction in iTTBK1 Tg and control animals in (**a**) Female or (**b**) Male animals. **c**, **d** Percent weight change from an individual animal’s initial weight while on DOX compared to their weight after 5 weeks of transgene expression in (**c**) Female or (**d**) Male animals. e-f Latency of iTTBK1 Tg and control animals to fall from an inverted grid as a function of length of transgene expression (weeks off DOX) in (**e**) Female or (**f**) Male animals. **g**, **h** Grip strength measurements in iTTBK1 Tg and control animals at 0, 2, 4, and 6 weeks off DOX using a dynamometer in (**g**) Female or (**h**) Male animals.

Additional file 3: Figure S3. Clasping, distance traveled in Open Field, and distance traveled in the center of the Open Field separated by sex. **a**, **b** Increasing hind limb clasping behavior was observed in iTTBK1 Tg mice in the weeks following transgene induction (mean ± SEM) in (**a**) Female or (**b**) Male animals. **c**, **d** Open field testing of control and iTTBK1 Tg mice. iTTBK1 Tg are hyperactive compared to controls in (**c**) Female or (**d**) Male animals. **e**, **f** Open field testing of control and iTTBK1 Tg mice, percentage of distance travelled in the center of the field versus the periphery in (**e**) Female or (**f**) Male animals.

Additional file 4: Figure S4. **a** Immunoblot of phosphorylated tau (pSer422) or phosphorylated TDP-43 (P-TDP-43) from control or iTTBK1 Tg brain lysate. **b**−**g** The CA3 region of the hippocampus was assessed in iTTBK1 Tg. Transgenic mouse strains with known accumulations of pathological tau (tg2652 , which express 1N4R human tau), or pathological TDP-43 (rNLS which express human TDP-43 with a defective nuclear localization signal), were used as positive controls [27, 28]. **b**, **c** Paired helical filament (PHF) and phosphorylated S202 and T205 tau (AT8) is present in tg2652 but not in iTTBK1 Tg animals. **d**, **e** Phosphorylated S422 tau is present in tg2652 but not in iTTBK1 Tg animals. **f**, **g** Phosphorylated S409 and S410 TDP-43 is present in rNLS but not iTTBK1 Tg animals. Phosphorylated tau or TDP-43 was similarly absent from forebrain, brainstem, and spinal cord of iTTBK1 Tg mice (data not shown). **h**, **i** NeuN staining of neurons in the hippocampus of control or iTTBK1 Tg mice.

Additional file 5: Figure S5. **a** TTBK1 expression in whole brain lysate subjected to sequential extractions in detergents of increasing solubilizing strengths. LS = low salt (soluble), TX = non-ionic detergent, SARK = ionic detergent, UREA = detergent insoluble fraction. **b** Representative immunostaining of GFAP near cerebellar Purkinje cells in (**b**) control or (**c**) iTTBK1 Tg animals. Scale bar = 50μm. **d** mRNA levels of mouse *Ttbk2* are unchanged between control or iTTBK1 Tg mice as assessed by qRT-PCR. Expression of *Ttbk2* was normalized to expression of an internal control gene *Ywaz* to generate relative expression levels, p > 0.9999.

## Data Availability

The datasets generated and analyzed during this study are included in this published article and its supplementary information files, or are available from the corresponding author on reasonable request.
